# Identifying and Engineering Genes for Parthenogenesis in Plants

**DOI:** 10.3389/fpls.2019.00128

**Published:** 2019-02-19

**Authors:** Kitty Vijverberg, Peggy Ozias-Akins, M. Eric Schranz

**Affiliations:** ^1^Biosystematics Group, Experimental Plant Sciences, Wageningen University and Research, Wageningen, Netherlands; ^2^Department of Horticulture, Institute of Plant Breeding, Genetics and Genomics, University of Georgia, Tifton Campus, Tifton, GA, United States

**Keywords:** apomixis, embryogenesis, embryo induction, *PsASGR-BabyBoom-Like* (*PsASGR-BBML*), doubled haploids, parthenogenesis, *Pennisetum*, *Taraxacum*

## Abstract

*Parthenogenesis* is the spontaneous development of an embryo from an unfertilized egg cell. It naturally occurs in a variety of plant and animal species. In plants, parthenogenesis usually is found in combination with *apomeiosis* (the omission of meiosis) and *pseudogamous* or *autonomous* (with or without central cell fertilization) endosperm formation, together known as *apomixis* (clonal seed production). The initiation of embryogenesis *in vivo* and *in vitro* has high potential in plant breeding methods, particularly for the instant production of homozygous lines from haploid gametes [*doubled haploids* (DHs)], the maintenance of vigorous *F1-hybrids* through clonal seed production after combining it with apomeiosis, reverse breeding approaches, and for linking diploid and polyploid gene pools. Because of this large interest, efforts to identify gene(s) for parthenogenesis from natural apomicts have been undertaken by using map-based cloning strategies and comparative gene expression studies. In addition, engineering parthenogenesis in sexual model species has been investigated via mutagenesis and gain-of-function strategies. These efforts have started to pay off, particularly by the isolation of the *PsASGR-BabyBoom-Like* from apomictic *Pennisetum*, a gene proven to be transferable to and functional in sexual pearl millet, rice, and maize. This review aims to summarize the current knowledge on parthenogenesis, the possible gene candidates also outside the grasses, and the use of these genes in plant breeding protocols. It shows that parthenogenesis is able to inherit and function independently from apomeiosis and endosperm formation, is expressed and active in the egg cell, and can induce embryogenesis in polyploid, diploid as well as haploid egg cells in plants. It also shows the importance of genes involved in the suppression of transcription and modifications thereof at one hand, and in embryogenesis for which transcription is allowed or artificially overexpressed on the other, in parthenogenetic reproduction. Finally, it emphasizes the importance of functional endosperm to allow for successful embryo growth and viable seed production.

## Introduction

### Parthenogenesis and Spontaneous Embryo Development

*Parthenogenesis* is the spontaneous development of an embryo from an *unfertilized* egg cell: *parthenos* = virgin, *genesis* = creation. It naturally occurs in a variety of plant and animal species, particularly in lower plants such as mosses and algae and species-rich invertebrate groups such as insects, nematodes, and crustaceans, but also in c. 10% of the fern and 1% of the flowering plant species, and as rare examples in vertebrates (Bell, 1982; Suomalainen et al., 1987; Asker and Jerling, 1992; Schön et al., 2009; Hand and Koltunow, 2014; Grusz, 2016). In plants, the egg cell develops within a *female gametophyte*, which is a multicellular organism that arises from a megaspore, a product of female meiosis. The female gametophyte is thus haploid (*1n*) and alternates with the diploid (*2n*) sporophytic generation after fertilization of the egg cell with a haploid male sperm cell (e.g., Niklas and Kutschera, 2010; Schmidt et al., 2015; Bowman et al., 2016). In more primitive plants, the mosses and ferns, female gametophytes are relatively large, free-living organisms and egg cells develop in special regions, the archegonia. In higher plants, the female gametophyte (also embryo sac) is highly reduced. Circa 70% of the angiosperm species produce female gametophytes of the *Polygonum*-type, which consists of seven cells only: two gametes, the egg cell and central cell, and five accessory cells, the two synergids and three antipodals (Maheshwari, 1950). Animals lack an intermediate organism similar to the female gametophyte. Here, the egg cell is a direct product of meiosis and, as such, similar to the megaspore.

Parthenogenesis usually occurs in combination with a mechanism that keeps or restores the *diploid chromosome* number, since haploid offspring are usually less fit or non-viable in nature. Depending on the mechanism involved, true or partial clones of the mother are produced. In angiosperms, one of two types of *apomeiosis* (*apo* = without) occur: *apospory*, in which the gametophyte develops directly from a sporophytic cell of the ovule, or *diplospory*, in which meiosis is omitted, restituted, or preceded by endoreplication in the megaspore mother cell (Nogler, 1984; Asker and Jerling, 1992). In both cases, true clones of the mother plant are formed given that in diplospory, chromosome restitution happens before crossing-over has initiated, and after endoreplication, copy- rather than sister-chromosome pairing occurs. However, there are reported exceptions of recombination in diplosporous apomicts, e.g., in dandelion (Malecka, 1965). Apomeiosis can also be facultative, in which part of the offspring is produced by sexual means. This is found in diplosporous species, e.g., *Erigeron* (Noyes, 2005), but particularly also in pseudogamous aposporous species in which ovule-derived embryo sacs develop next to the reduced embryo sac and autonomous versus the sexually derived embryo are competing for resources, e.g., *Paspalum* (Ortiz et al., 2013). Similar mechanisms of apomeiosis exist in parthenogenetic animals, although here more often meiosis still occurs, involving either haploid offspring or restoration of diploidy through various mechanisms (Avise, 2008).

Successful embryo development depends on a third factor, the nutrition of the embryo. In angiosperms, the embryo is nourished by the endosperm, a tissue that in sexual individuals arises via fertilization of the central cell. The process of double fertilization in which the egg cell and central cell each are fertilized by one of two clonal sperm cells is unique to flowering plants (see for a review, e.g., Dresselhaus et al., 2016). In most apomictic species, endosperm development is *pseudogamous*, requiring fertilization of the central cell, whereas in a minority of apomictic species, the endosperm develops *autonomously*. In both cases, the usual maternal (*m*) *versus* paternal (*p*) genome ratio of *2m : 1p* in the endosperm might be altered, which can severely affect seed development in many plant species (Scott et al., 1998; Autran et al., 2005; Kradolfer et al., 2013). Apomicts evolved different adaptations to overcome this requirement, e.g., in pseudogamous panicoid grasses only four nuclei comprise the aposporous embryo sac with predominantly unreduced, uni-nucleate central cells fertilized by a reduced sperm (Ozias-Akins, 2006). In animals, embryo nutrition is provided by the mother in one of many ways, without the need for a second fertilization event. In some parthenogenetic animal species, however, embryo development needs activation by a sperm without the fusion of gametes, known as *gynogenesis* or *sperm-dependent parthenogenesis*. Parthenogenesis and apomeiosis together, combined with either *pseudogamous* or *autonomous* endosperm formation, is defined as *apomixis* (*sensu stricto*) or *agamspermy*, the clonal seed formation (reviewed by, e.g., Pupilli and Barcaccia, 2012; Hand and Koltunow, 2014; Conner and Ozias-Akins, 2017).

Parthenogenesis is one form of *apogamy* and is sometimes also used in a wider sense, including spontaneous embryo development from (a) *gametophytic cell*(s) other than the egg cell, which is particularly common in lower plants (Asker and Jerling, 1992). *Apogamy sensu lato* includes, in addition, the spontaneous development of an embryo from a *sporophytic cell*, known as *somatic embryogenesis*. This process lacks the development of an embryo sac, endosperm, and seed coat. A classic example of somatic embryogenesis is the spontaneous embryo formation at leaf margins in *Kalanchoë* spp. (Garcês et al., 2007). A particular form of it is *adventitious embryony* or *polyembryony* in which the embryo(s) develop(s) from a sporophytic cell of the ovule (Nogler, 1984). Another special form is *in vitro embryogenesis* in which embryos develop ex-planta usually from microspores (pollen) or, less frequently, female gametophytic cells (*gametophytic embryogenesis*), or protoplasts, leaves, hypocotyls, or other plant tissues (*sporophytic embryogenesis*), often indirectly via the formation of a callus (Horstman et al., 2017). The reprogramming to progress into *embryogenesis* occurs under the influence of external stimuli such as hormones, heat stress, or overexpression of particular TFs (Hand et al., 2016; Ikeuchi et al., 2016). Although successful in a range of species, many species or particular genotypes can be (very) recalcitrant to *in vitro embryogenesis* and unable to produce embryos with any of the known stimuli (Ochatt et al., 2010; Soliìs-Ramos et al., 2012). Identifying a gene that is able to induce parthenogenesis particularly in these recalcitrant species and genotypes would be very valuable as a tool in plant breeding.

**FIGURE 1:**
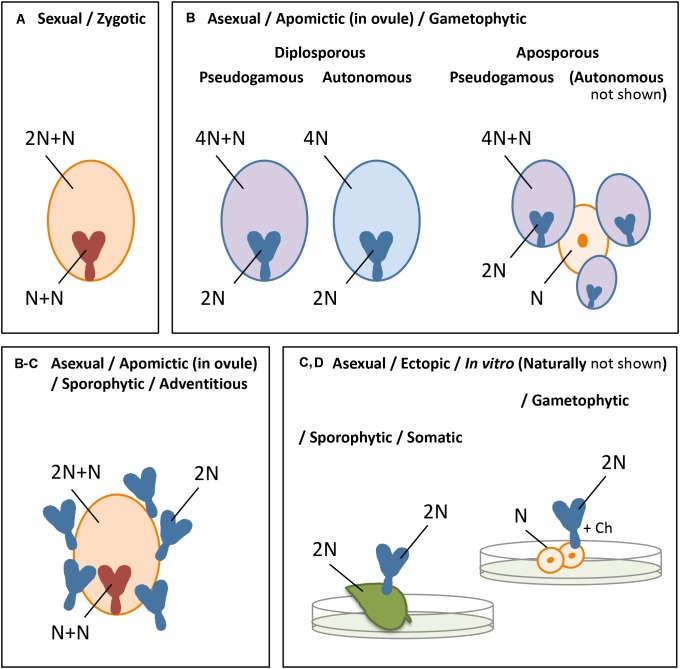
Summary of the different forms of *embryogenesis* in plants, showing the embryo and endosperm originated from a mature embryo sac **(A,B)** or the embryo ectopically **(C,D)** after sexual **(A)** or asexual **(B–D)** reproduction, with orange indicating the sexual process, blue the asexual or apomictic process, pink apomictic reproduction with fertilization of the central cell, and N = chromosome set after reduction division: **(A)** *zygotic embryogenesis*, involving chromosome reduction (N) and gamete fusion (N+N for the embryo, 2N+N for the endosperm), **(B)** *apomictic embryogenesis*, occurring in the ovule, either *gametophytic apomixis* in which an embryo sac arises from an unreduced megaspore (diplospory) or sporophytic cell of the ovule, usually adjacent to a sexually derived spore or developing embryo sac (apospory), and parthenogenetic (spontaneous) embryo development and autonomous (spontaneous) or pseudogamous (after fertilization of the central cell) endosperm formation, or *sporophytic apomixis* in which the embryo arises directly from a sporophytic cell of the ovule, often as polyembryony and alongside a sexually derived embryo and endosperm **(C)** *somatic*/*sporophytic embryogenesis*, involving ectopic embryo development from sporophytic cells, and **(D)** *gametophytic embryogenesis*, idem from a gametophytic cell. The latter two **(C,D)** omit the formation of an embryo sac, endosperm, and a seed coat, and occur naturally, for example, from leaf margins or ovular cells **(C)**, gametophytic tissue in lower plants or, e.g., a synergid **(D)**, but are particularly known from *in vitro embryogenesis* in which embryos are formed in culture, after external induction, particularly from protoplasts, leaf, the hypocotyl or other plant tissues **(C)**, or microspores **(D)**.

*The different forms of embryogenesis* are summarized in Figure 1. In this review, we focus on parthenogenesis in the strict sense, concerning the spontaneous development of an embryo from an unfertilized egg cell, and in flowering plants. Nevertheless, the *induction of embryo development* from any other cell or tissue may include commonalities with this process in the plant egg cell and, where overlapping, this will be considered in addition.

### Historical Aspects of Parthenogenesis

The use of “*Parthenogenesis*” to denote asexual reproduction in plants followed its use in insects, as by *Owen (1849): “On parthenogenesis, or the successive procreating individuals from a single ovum”* (1849) and von Siebold (1856): “*Wahre Parthenogenesis bei Schmetterlingen und Biene, ein Beitrag zur Fortpflanzungsgeschichte der Thiere*” (1856). In fact, *apomixis* rather than parthenogenesis is meant here, also including *apomeiosis* and endosperm development. The recognition that some plant species are capable of asexual reproduction came only two decades after the universality of “the law of sexual reproduction” was established, realizing that this also holds for plants (c. 1830) (Nogler, 2007). Many discussions and numerous investigations in the two prior centuries preceded recognition (reviewed by Bergsma, 1857). Most of these studies included *dioecious* plant species, particularly *Cannabis, Mercurialis, Spinacia, Curcurbits, Silenes*, and *Ricinus*, and some monoecious species with separate male and female flowers, e.g., maize. However, due to the state of knowledge at that time and limited technical possibilities, all studies violated to a certain degree one or both of two essential conditions: (1) complete isolation of the plants and (2) exact observations (Bergsma, 1857). Regarding (1), some studies were done even before the discovery of the pollen or knowledge of its function (e.g., Camerarius, 1694), and (2), particularly the occasional formation of male organs in female flowers remained unrecognized. A relationship between asexual seed production and either annual plants or monoecious species has also been suggested (among the early investigators: Spallanzani, L., c. 1770–1785, Bernhardi, J., c. 1834–1839, Lecoq, H., c. 1858–1867, and Naudin, C., c. 1861–1867; see Bergsma, 1857). Ultimately, only one of these reports of asexual seed production was confirmed and is now considered as its first proof, namely, that *Coelebogyne ilicifolia* (presently: *Alchornea ilicifolia*; Euphorbiaceae) produces perfect seeds without any apparent action of pollen (Smith, 1841). After better fixing and staining methods became available, this example of asexual seed was revealed to result from polyembryony rather than parthenogenesis (Strasburger, 1877). It took another two decades to confirm that *true* parthenogenesis indeed exists in angiosperms, verified on the basis of careful observations in *Antennaria alpina* (Juel, 1898). Apomeiosis in this species involves the omission of meiosis in the megaspore mother cell, followed by *two* mitotic divisions resulting in four unreduced spores, now known as the *Antennaria*-type of diplospory. Subsequently, parthenogenesis was proven to occur in other species, including *Taraxacum* and *Hieracium* (Murbeck, 1904), and involve additional modes of apomeiosis (Juel, 1906; Rosenberg, 1906; reviewed by Nogler, 2007). According to our current knowledge, ∼400 species from different plant families are able to produce seeds without fertilization, and apomixis evolved numerous times in plants (Carman, 1997; van Dijk and Vijverberg, 2005). This suggests that parthenogenesis likely also relies on more than one genetic mechanism.

## Egg Cell Arrest and the Trigger for Embryogenesis

In parthenogenetic reproduction *egg cell arrest*, as is found in sexual reproduction prior to fertilization, is absent or strongly reduced. Cytological investigations indicate a short period of egg cell arrest at least in some apomictic species, followed by precocious (before anthesis) embryo development (Nogler, 1984), e.g., in dandelion (van Baarlen et al., 2002) and the wild relative of wheat, *Tripsacum dactyloides* (Grimanelli et al., 2003). In sexual plants at the end of female gametophyte patterning (see for a review Tekleyohans et al., 2017), the mature egg cell is characterized by highly condensed *repressive chromatin* and a relatively *quiescent transcriptional* state (Garcia-Aguilar et al., 2010; Pillot et al., 2010). This is hypothesized to be necessary for attaining *totipotency* in the zygote and early embryo (Baroux and Grossniklaus, 2015). After fertilization and karyogamy, structural changes in the chromatin are necessary to enable access to the DNA for transcription and the replication machinery. Whether the egg cell in apomicts also undergoes a (brief) period of chromatin repression and transcriptional silencing before embryogenesis initiates is yet unknown, but likely if indeed needed to obtain totipotency. Parthenogenesis may then involve factors that are responsible for the spontaneous de-repression of chromatin and activation of transcription. Alternatively, the egg cells in apomicts bypass a chromatin repressive and transcriptionally silent state and need reprogramming. In this context, it is interesting to know the chromatin state in initial cells of embryogenesis other than egg cells, e.g., in somatic embryogenesis, to search for parallels. In any case, factors that are involved in *chromatin-remodeling* and *transcriptional regulation* are candidates to play a role in the parthenogenetic pathway.

In egg cell arrest and embryo development, a role for signals from the *surrounding tissue* is indicated, particularly from the companion cells, the *central cell* in the mature gametophyte and *endosperm* in the developing seeds (Grossniklaus, 2011). In sexual plant reproduction, the central cell also arrests until fertilization, but shows chromatin that is depleted from repressive marks and displays a more active transcriptional competence (Baroux and Grossniklaus, 2015). This allows for the expression of *maternal alleles* and TEs, the latter thought to serve the production of 24 nucleotide siRNAs to reinforce silencing of TEs in the egg cell (Ibarra et al., 2012; Roche et al., 2016; Martinez and Köhler, 2017). The former, the expression of maternal alleles, may contribute to the differential expression of maternally and paternally inherited alleles in the early endosperm after fertilization, as is also the results of imprinted genes (Wang and Köhler, 2017). In many species, the endosperm is, therefore, sensitive to a *maternal* to *paternal* dosage, and deviations from this lead to endosperm failure and embryo arrest (Scott et al., 1998; Kradolfer et al., 2013). Silencing of TEs in the egg cell by small RNAs from the *surrounding nucellar tissue* is also reported. A study in *Arabidopsis* shows reactivation of TEs in the egg cells of plants that are mutant for *ARGONAUT*9 *(AGO*9*)*, a small RNA binding protein of the RNA Induced Silencing Complex (RISC) (Olmedo-Monfil et al., 2010). Another study shows higher overall transcription levels in early embryos of parthenogenetic *Tripsacum x* maize hybrids as compared to embryos of sexual maize, supporting reduced silencing under parthenogenetic conditions (Garcia-Aguilar et al., 2010). Taken together, findings suggest that: (1) dedicated (TE-)silencing pathways, involving companion cells and surrounding ovular tissue, result in dynamic patterns of transcriptional suppression in the egg cell, and (2) the *m : p* balance in the endosperm is important for proper functioning of the endosperm, which in turn is essential for embryo survival. They imply that changes in the accompanying cells and, as a result, in the communication to the egg cell, for example, changes in genes involved in DNA (de-)methylation or small RNA pathways, may have evolved in parthenogenesis.

In the sexual model species Arabidopsis, *central cell arrest* requires control by the PRC2, an evolutionarily conserved complex that is involved in the suppression of development via the regulation of epigenetic modulation (reviewed by Mozgova and Hennig, 2015; Wang and Köhler, 2017). The PRC2 maintains the repressive state of its target genes by preserving the tri-methylation of the N-terminal tail of histone H3 on lysine 27 (H3K27me3), a mark of transcriptional silencing. Different PRC2s exist, with the one involved in seed development containing the fertilization independent seed (FIS)-class proteins: *MEDEA* (*MEA*) (Grossniklaus et al., 1998), *FIS*2 (Luo et al., 1999), *FERTILIZATION-INDEPENDENT ENDOSPERM* (*FIE*) (Ohad et al., 1999), and *MULTICOPY SUPPRESSOR OF IRA*1 (*MSI*1) (Köhler et al., 2003a). Mutations in one of the *FIS*-class genes result in autonomous endosperm formation, showing diploid nuclei and development until cellularization (Chaudhury et al., 1997). Mutations in *MSI*1 result in spontaneous embryo development in addition, although, with early embryo abortion up to the c. 20-cell stage (Guitton and Berger, 2005). These non-viable, haploid embryos express molecular markers and polarity similar to the diploid wild-type embryos produced by fertilization. Mutants of the *FIS*-class genes *mea* and *fis*2 also rarely show embryo-like structures (Chaudhury et al., 1997). Since the penetrance of *FIS*-mutants on autonomous endosperm development is highest for *msi*1 (Köhler et al., 2003a), possibly the egg cell is able to undergo spontaneous development also in other *FIS-*mutants, but with lower penetrance (Chaudhury et al., 1997). Later studies showed that the functional requirement of the *FIS*-PRC2 could be bypassed by increasing the maternal genome dosage in the endosperm (Kradolfer et al., 2013), and that the *FIS*-PRC2 functions in the repression of maternal alleles of paternally expressed imprinted genes (reviewed in Wang and Köhler, 2017). The authors proposed that the *FIS*-PRC2 evolved concomitantly with sexual endosperm and the angiosperms. This is particularly interesting in the context of apomixis, in which the ability to reproduce sexually is lost or modified and the maternal genome dosage in the endosperm is usually increased in pseudogamous apomicts and unique in autonomous apomicts. Apomictic species may thus have become independent from the *FIS*-PRC2, either because it has a (relatively) modified expression or they evolved changed requirements for it. Unraveling this changes in more detail may give clues for parthenogenetic reproduction.

Despite the great discoveries discussed above, the precise molecular *mechanism(s)* by which the egg cell achieves its competence and is activated for embryogenesis is still unknown. In animals, early embryogenesis mainly depends on maternal genetic information deposited in the egg cell before fertilization (Tadros and Lipshitz, 2009; Eckersley-Maslin et al., 2018). During the MZT and ZGA, maternal transcripts are degraded and zygotic ones synthesized. In flowering plants, the large cytoplasm of the egg cell also allows for the deposition of maternally derived molecules. Single cell type transcriptome analyses confirmed that the egg cell of the dicot *Arabidopsis* (Wüst et al., 2010) and monocots rice (Anderson et al., 2013) and maize (Chen et al., 2017) is stocked with RNAs, proteins and other molecules that support embryogenesis upon activation. Circa 30–40% of the total number of genes are expressed in the egg cell, a percentage not notably lower than in other (gametic) cells. Several evidences suggest that embryogenesis in plants also mainly relies on maternal transcripts (Autran et al., 2011), although paternal contribution soon after fertilization is also reported (Del Toro-De León et al., 2014; Anderson et al., 2017), and hypothesized to trigger embryogenesis of fertilized egg cells (Khanday et al., 2018). If particularly or solely maternal transcripts are involved in the initiation of embryogenesis and MZT, parthenogenetic embryo development might be similar to that in sexual reproduction. However, if paternal factors are involved in addition, alternatives for their need should have been evolved in parthenogenesis, e.g., by activation of usually silent maternal transcripts. Transcripts over-represented in the egg cells of *Arabidopsis* include TF-families, particularly those of type I MADS domain, RWP-RK domain, and reproductive meristem (Wüst et al., 2010). In addition, *PIWI/ARGONAUTE/ZWILLE* (*PAZ*) domain encoding genes are upregulated, supporting a role for epigenetic regulation through small RNA pathways, and the *AUXIN RESPONSE FACTOR* 17 (*ARF*17) is enriched, suggesting the involvement of auxin. An interesting recent finding that highlights the importance of auxin in embryogenesis regulation is the identification of an auxin-response network that suppresses embryo development from the suspensor in *Arabidopsis* (Radoeva et al., 2018). In rice and maize egg cells, TFs are also over-represented, as are genes involved in transcriptional regulation and nucleic acid binding (Anderson et al., 2013; Chen et al., 2017). A comparative transcriptome analysis between egg cells and zygotes in maize shows ZGA to involve c. 10% of the genome (Chen et al., 2017). Particularly genes that encode transcriptional regulators are activated in ZGA and chromatin assembly is modified, while the egg cell becomes primed to activate the translational machinery. In summary, data show that a range of molecules known to play a role in development are stored in the egg cell and ready for use in embryogenesis. They suggest that only a trigger is needed to release the repressive state and activate transcription and translation in order to initiate this.

In vertebrate egg cells, different evidence suggests that the key trigger for egg cell activation is a rise in intercellular *Ca*^2+^, initiated by the fertilizing sperm and responsible for all further downstream reactions (Horner and Wolfner, 2008; Machaty, 2016). An increase of internal *Ca*^2+^ is also detected in zygotes of maize (Digonnet et al., 1997; Antoine et al., 2001) and wheat (Pónya et al., 2014) in *in vitro* fertilization experiments after the fusion of the gametes. Subsequently, cell wall material is formed, likely representing a block to polyspermy. A role for *Ca*^2+^ in cell–cell communication during plant fertilization was suggested by detecting a *Ca*^2+^ maximum at pollen tube rupture (Dresselhaus and Franklin-Tong, 2013). A short *Ca*^2+^ transient in both the egg and central cell was associated with pollen tube burst and sperm cell arrival, while a second extended *Ca*^2+^ transient solely in the egg cell was correlated with successful fertilization (Denninger et al., 2014). Although rising upon the fusion of gametes, a *Ca*^2+^ rise alone apparently is insufficient to trigger parthenogenesis in plants. In some parthenogenetic organisms of other kingdoms, such as insects, stimuli imparted to the egg cell during ovulation or egg-laying, or non-sperm-based signals, e.g., a change in ionic strength or pH, can trigger egg cell activation (Horner and Wolfner, 2008). *In vitro* protocols for *DH*-production make use of external stimuli such as a change in ion concentration or other abiotic stress factors to induce embryogenesis in micro- and megaspores (Germanà, 2006; Belogradova et al., 2009; Islam and Tuteja, 2012; Hand et al., 2016). However, these external stimuli are not generally applicable, and despite being successful in some species and genotypes others can be completely recalcitrant to such triggers. Nevertheless, they suggest that the breaking of egg cell arrest and/or release of the repressive chromatin and transcriptional silent state may (also) involve a change in (internal) physiology, particularly involving *Ca*^2+^. Searching for factors that underlie such changes likely aid in defining the molecular basis of parthenogenesis.

In summary, data show that egg cells in sexually reproducing species undergo a period of arrest that goes together with condensed, repressive chromatin and silenced transcription, and an egg cell that is stocked with molecules ready for use in embryo development. The molecular mechanism(s) or trigger by which the egg cell is activated and embryogenesis initiates is yet unknown, but results suggest the involvement of factors that release the chromatin repressive and transcriptionally silent state, e.g., genes involved in (de-)methylation, small RNAs, and hormones or a change in physiology. Particularly the inactivation or modification of the *FIS*-PRC2 may play a role in these changes. Parthenogenetic egg cells lack arrest or arrest for only a (very) short period, and it is unknown whether this implies that chromatin repression and transcriptional silencing are also omitted. Since a quiescent state is hypothesized to be necessary to attain totipotency in the zygote, probably this state occurs also in parthenogenetic eggs, but only for a (very) short period. In any case, factors that are involved in chromatin-remodeling or transcriptional regulation are likely candidates in the parthenogenesis developmental pathway.

## Genetic Control of Parthenogenesis in Apomicts and Its Independency From Apomeiosis and Polyploidy

Parthenogenesis in angiosperms has long been thought to be a process that was initiated by apomeiosis and unknown to occur independently from it. Also the genetic control of parthenogenesis and apomeiosis was long assumed to rely on a single (master) gene or one locus with tightly linked genes (Mogie, 1992). However, some of the former genetic models (Richards, 1970; Nogler, 1984; Asker and Jerling, 1992) and more recent genetic mapping and other studies (reviewed by Vijverberg and van Dijk, 2007; Hand and Koltunow, 2014), showed that parthenogenesis is able to segregate from apomeiosis. Among the first evidence for this came from artificial crosses in the common dandelion (*Taraxacum officinale*), using diploid sexual female x triploid apomictic male crosses, resulting in small amounts of diploid, triploid, and tetraploid hybrid offspring, corresponding to fertilization of the haploid egg cell with a haploid, diploid, or triploid sperm, respectively (Tas and van Dijk, 1999; van Dijk et al., 1999). All tetraploid and some triploid hybrids showed spontaneous seed formation, thus displaying all apomixis elements. The other triploid hybrids produced (near-) diploid (type-A; three plants), triploid (type-B; four plants), or tetraploid (type-C; two plants) offspring after pollination with haploid pollen. Additional cytological investigations (van Baarlen et al., 2002) and molecular marker studies (van Dijk et al., 2003) showed that type-B hybrids were diplosporous, parthenogenetic, and lacked endosperm autonomy, while type-C hybrids were diplosporous, with autonomous endosperm formation, but lacking parthenogenesis. Apart from demonstrating that apomeiosis, parthenogenesis, and also endosperm autonomy, were inherited independently from each other, these results established that parthenogenesis functions independently from endosperm autonomy and *vice versa* in dandelion. Some offspring of the type-B triploids originated from parthenogenetic development (2*n + 0* embryos), whereas the remainder resulted from fertilization of the egg cell (2*n + n*) suggesting incomplete, ∼2/3rd penetrance of parthenogenesis in these hybrids (van Dijk et al., 1999). Apparently, the usual precocious development of the embryo, as occurs in full apomicts, was disturbed, possibly as a result of the separation of parthenogenesis from apomeiosis or from modifiers or enhancers. All type-A triploids and the diploid hybrids gave rise to diploid offspring only after pollination with haploid pollen, suggesting that they were *true* sexuals. The absence of apomeiosis in diploid hybrids was later confirmed by the absence of a linked microsatellite marker in progeny from a similar cross (van Dijk et al., 2009). It was suggested to be the result of a genetic load associated to long-term asexual reproduction that becomes apparent and lethal in haploid gametes. This would then possibly also hold for parthenogenesis. Although presumed, the absence of parthenogenesis in the type-A triploids and diploid hybrids and the independent acting of parthenogenesis from apomeiosis were not explicitly demonstrated.

Shortly afterward, the separate inheritance of apomeiosis and parthenogenesis was confirmed in another diplosporous Asteraceae, *Erigeron annuus* (Noyes, 2000; Noyes and Rieseberg, 2000). In a follow-up, Noyes et al. (2007) re-confirmed the existence of one locus for diplospory (*D*) and a separate locus for parthenogenesis *and* endosperm autonomy (*F*), and showed that spontaneous development (*F*) occurred in the presence as well as absence of *D*, although, with early embryo abortion in the meiotic context. Parthenogenesis could thus act as an *embryogenesis inducer* in the absence of apomeiosis, whereas a possible role in *embryo growth* needed verification. All offspring investigated in these studies were triploid, implying imbalanced meiosis, resulting in aberrant chromosome numbers in most of the egg cells. Apparently, parthenogenesis can act in an *aneuploid context*, although with early embryo arrest. More recently, Noyes and Wagner (2014) showed that in autonomously produced *di-haploid* offspring from a tetraploid synthetic apomict (genotype: *Dddd/Ffff*) with incomplete, c. 50% penetrance of diplospory, *D* segregated 1:1 and parthenogenesis (*F*) was present in all (genotypes: *Dd/Ff* and *dd/Ff*). This confirmed that parthenogenesis is able to act and give rise to viable offspring independently from apomeiosis, at least, in a di-haploid context. Due to the absence of the genotypes *Dd/ff* and *dd/ff* among the offspring obtained without pollination, one could infer that the functioning of *F* is based on its presence and expression in the female gametophyte rather than on signals from the surrounding sporophytic maternal tissue. The study also mentioned rare, spontaneous development of seeds by some of the di-haploid parthenogenetic plants, that germinated normally and grew-out into haploid plants up to flowering (Noyes and Wagner, 2014). This showed that parthenogenesis is able to function in a haploid context, although, with (very) low efficiency. The low haploid survival rate is likely a result of recessive-lethal selection against the parthenogenesis locus, as was suggested in one of the previous studies in *Erigeron* for the absence of apomixis elements from diploid offspring (Noyes and Rieseberg, 2000).

Currently, the separate inheritance of apomeiosis and parthenogenesis is confirmed for most apomictic model species, including aposporous and diplosporous apomicts and monocots as well as dicots, as is implied by, e.g., a cytological study in *Poa pratensis* that shows two aposporous, non-parthenogenetic individuals (Albertini et al., 2001); flow cytometric analysis in *Hypericum perforatum*, indicating parthenogenetic development in 10% of the reduced, di-haploid offspring in the presence of pseudogamous endosperm formation (Barcaccia et al., 2006); idem in the guinea grass *Panicum maximum*, resolving eight different reproductive pathways of seed development (Kaushal et al., 2008); a gamma deletion mapping study in *Hieracium caespitosum*, showing one locus for *Loss-Of-Apospory* (*LOA*) and one for *Loss-Of-Parthenogenesis* (*LOP*) (Catanach et al., 2006); comparative mapping studies in *Pennisetum squamulatum* versus *Cenchrus ciliarus*, resolving one aposporous recombinant that lacks parthenogenesis (Conner et al., 2013); and backcrossing experiments in *Allium*, resulting in diplosporous individuals with and without parthenogenetic development (Yamashita et al., 2012). The independent inheritance of endosperm autonomy from parthenogenesis is also supported by other studies, e.g., in *Hieracium*, the factor *AutE* was found to function independently from apospory and parthenogenesis (Ogawa et al., 2013). Mapping studies support the dominant monogenic/-locus inheritance of both, apomeiosis and parthenogenesis. The usual co-segregation of the apomixis elements apparently is the result of their location in complex, repeat, and transposon-rich, non-recombining and in some species hemizygous genomic regions, particularly illustrated by the >50 Mbp long ASGR in *P. squamulatum* (Akiyama et al., 2004) and the Apomixis Controlling Locus in *Paspalum simplex* (Labombarda et al., 2002). This co-segregation likely evolved as a prerequisite for each of the apomixis elements alone to survive, since their separate occurrence will be untenable in the long-term due to their creation of plant lines with accumulating increasing or decreasing ploidy levels (Asker and Jerling, 1992).

Recently, the long-term studies in natural apomicts paid off by resolving the *BABY BOOM-Like (BBML*) gene in *P. squamulatum* as a candidate gene for parthenogenesis (Conner et al., 2015; next paragraph). Transgenes of *PsASGR-BBML* were able to induce parthenogenesis in the tetraploid sexual relative *P. glaucum*, supporting their function in di-haploid eggs. *PsASGR-BBMLpromoter-GUS* analysis provided evidence for the expression of parthenogenesis in the egg cells of *P. glaucum*, where GUS expression was observed from 1 day before anthesis and in the post-fertilization developing embryos. These observations confirmed the function of a parthenogenesis gene on the basis of its presence and expression in the egg cell, rather than the companion central cell and/or surrounding sporophytic tissue. In a more recent study, it was shown that *PsASGR-BBML* transgenes were also able to induce parthenogenesis in haploid eggs of sexual diploid rice and maize (Conner et al., 2017). This clearly demonstrated that parthenogenesis is fully functional also in the haploid context, and supports that the usual absence of parthenogenesis from haploidy is likely a result of recessive-lethal selection against associated flanking genomic regions.

In summary, studies in natural apomicts show that parthenogenesis usually co-segregates with apomeiosis, but is able to segregate and function independently from it. Parthenogenesis can also segregate and act independently from autonomous endosperm formation. Most studies indicate monogenic/-locus, dominant inheritance of parthenogenesis. It functions normally in di-haploid egg cells, and is able to induce embryogenesis in aneuploid eggs, although, with early embryo abortion. Parthenogenesis is virtually absent from reduced, haploid plant egg cells, but rare observations of its functioning in haploid eggs have been reported for di-haploid parthenogenetic *Erigeron* (see above) and a diploid apomictic *Hieracium* plant (Bicknell, 1997). The isolation of the parthenogenesis inducing *PsASGR-BBML* gene, and the demonstration of its function as a transgene in haploid eggs from *Pennisetum*, rice, and maize, confirms that parthenogenesis is able to act independently from polyploidy. It suggests that the absence of the trait from haploidy is likely explained by a genetic load in linked genomic regions. Finally, the results support the gametic presence and expression of parthenogenesis rather than non-cell-autonomous signaling from companion cells or surrounding, sporophytic tissue.

## Candidate Genes for Parthenogenesis

### The *PsASGR-BabyBoom-Like* Gene

A *BABY BOOM (BBM)-Like* gene was discovered in the natural apomictic grass *P. squamulatum* while skim-sequencing bacterial artificial chromosome clones that were linked to the ASGR (Conner et al., 2008). *BBM* genes were originally identified in *Brassica napus* (Boutilier et al., 2002) and shown to induce somatic embryogenesis in *B. napus* and *Arabidopsis* upon ectopic expression. They are part of a large gene family characterized by the *APETALA 2/ETHYLENE RESPONSE FACTOR* (*AP*2/*ERF*) DNA-binding domain (Jofuku et al., 1994; Riechmann and Meyerowitz, 1998). This TF-family of almost 150 members in *Arabidopsis* (Sakuma et al., 2002) and 157 members in rice (Nakano et al., 2006) is divided into groups of genes containing either one or two *AP*2 domains. The one-domain *ERF-like* genes typically are involved in biotic or abiotic stress response whereas the two-domain *AP2-like* genes function in growth and development (Floyd and Bowman, 2007). *BBM* genes along with *AINTEGUMENTA* (*ANT*) and *PLETHORA* (*PLT*) genes belong to the *AINTEGUMENTA-Like* (*AIL*) subclade within the eudicot*ANT* (eu*ANT*) class of *AP*2/*ERF* DNA-binding domain genes, all of which function during embryogenesis (Horstman et al., 2014). *PsASGR-BBML* protein sequence is most similar to other *BBML* genes from related apomictic species: *Cenchrus ciliaris* and *Pennisetum* spp., but also to *BBM* genes in *Setaria italica* (foxtail millet) and *Oryza sativa* (rice), and yet more distantly related to *BBM* genes in *Zea mays* and *Sorghum bicolor* (El Ouakfaoui et al., 2010; Conner et al., 2015). It is surprising that the rice *BBM*1 is more closely related to *PsASGR-BBML* than to any *BBM* copy in maize and sorghum, given that rice and the panicoid grasses that include *Pennisetum*, sorghum, and maize, diverged from one another around 60 million years ago.

None of the *AIL* genes in *Arabidopsis* is expressed in pre-fertilization gametic cells during sexual reproduction (Horstman et al., 2014). On the other hand, after transformation to sexual *P. glaucum* (pearl millet), the *PsASGR-BBML* gene was shown to be expressed in egg cells prior to fertilization and to be sufficient for the initiation of embryos in the absence of fertilization (Conner et al., 2015). Since tetraploid pearl millet was the transgenic background, parthenogenetic development of the reduced eggs gave rise to diploid progeny. A second cycle of parthenogenesis resulted in true haploids, which expectedly were sterile. Sterile haploids also were derived through parthenogenesis of reduced egg cells in rice and maize (Conner et al., 2017). Among these three transgenic cereals, it was demonstrated that both the native *PsASGR-BBML* promoter and an egg-cell-specific *Arabidopsis* promoter (DD45; Steffen et al., 2007) provided the appropriate temporal regulation to enable fertilization-independent embryo formation. Mature haploid seed formation was irregular possibly as a result of asynchronous embryo-endosperm development. This first demonstration of parthenogenesis gene function opens the door for synthesizing apomixis in cereal crops once the capacity to produce unreduced gametes at high frequency is installed.

Interestingly, it was recently found that a wild-type rice *BBM*1 (*Os-BBM*1) transgene under an *Arabidopsis* egg-cell-specific promoter (DD45) was also able to initiate embryogenesis in rice egg cells without fertilization (Khanday et al., 2018). This supported the close relationship of *PsASGR-BBML* with *Os-BBM*1 and the functionality of the associated AP2-*like* domain in parthenogenesis rather than an evolved novel capability in functional domains. Most interestingly, it was shown that *Os-BBM*1 lacks expression in the egg cells of rice, but is expressed in sperm cells, whereas only male *BBM*1-transcripts are expressed in early zygotes. This suggests the requirement of fertilization in embryogenesis for the transmission of male-genomic factors that are maternally silenced. It would imply that in parthenogenesis, an essential, normally maternally imprinted gene, may have become maternally expressed. This is supported by another interesting recent study that shows maternal expression of the normally imprinted gene *PHERES*1 (*PHE*1; Köhler et al., 2003b) in apomictic *Boechera* (Kirioukhova et al., 2018), as is further discussed in Section “The *FIS-PRC*2 and *RETINOBLASTOMA RELATED*1”. It nicely brings together the many studies that indicate the involvement of diverse repression mechanisms in egg cell arrest and the associated factors that putatively play a role in the release of this repression (see section “Egg Cell Arrest and the Trigger for Embryogenesis” above).

### The “Salmon System” in Wheat

Investigations in the context of parthenogenesis were done in the “Salmon system” of wheat (*Triticum aestivum*) already some decades ago. This system was developed for use in haploid production after it was recognized that transfer of the nucleus of the sexual cultivar “Salmon” to cytoplasm of the grass genus *Aegilops* resulted in lines that were capable of autonomous embryo development (Tsunewaki and Mukai, 1990). In the “Salmon” line, the short arm of chromosome 1B of wheat has been replaced by the short arm of chromosome 1R of rye. Tsunewaki and Mukai (1990) concluded that besides a cytoplasmic *Restorer of fertility* (*Rfv1*) factor, two nuclear genes were involved in spontaneous embryo development: the inducer gene *Parthenogenesis gain* (*Ptg*) that is under sporophytic control, and the suppressor gene *Suppressor of parthenogenesis* (*Spg*) that is under gametophytic control. These two genes were concomitantly exchanged with the chromosome 1 arm. Two other researchers came to the same conclusion in a 1B/1R-translocation system in durum wheat (Hsam and Zeller, 1993). To improve the system for *in vivo* investigation of parthenogenesis, three isogenic homozygous lines were produced, the male fertile sexual line *Ae. aestivum*-Salmon (*a*S), and the male sterile parthenogenetic lines *Ae. caudata*-Salmon (*c*S) and *Ae. kotschyi*-Salmon (*k*S) (Matzk et al., 1995). Comparative protein analysis from ovary extracts of these three lines resolved one protein that was uniquely expressed in the two parthenogenetic lines from 3 days before and during anthesis. This protein, P115.1, was characterized as a α-tubulin polypeptide. Tubulin α-chains are the major constituent of microtubules and function in GTP-binding and, regarding their function, could possibly also be a result of parthenogenesis rather than its cause. Further studies on isolated egg cells from the three isogenic lines and a common wheat line indicated that parthenogenetic development is independent from ovary-derived signals (Kumlehn et al., 2001). This encouraged the researches to focus on the egg cells and construct cDNA libraries from them. Analysis of these libraries delivered a number of egg cell specific candidates among which were RWP-RK domain (*RKD*)-containing TFs. These were subject to later studies in *Arabidospis* (Köszegi et al., 2011; Tedeschi et al., 2017) and *Marchantia polymorpha* (Rövekamp et al., 2016; Koi et al., 2016) (see next paragraph). Some eggs of the parthenogenetic lines showed a second nucleolus, a characteristic of zygotes isolated from sexual lines (Naumova and Matzk, 1998; Kumlehn et al., 2001). Together, the results showed that parthenogenesis apparently is an inherent property of the egg cell and not the surrounding tissue and is able to establish zygotic competence in the absence of fertilization.

### RWP-RK Domain (*RKD*)-Containing Transcription Factor

Encouraged by their identification in wheat egg cell cDNA libraries (Kumlehn et al., 2001; former paragraph) *RKD*-TF homologs were searched for and investigated in *Arabidopsis thaliana*, which resolved a total of five *AtRKD*s (Köszegi et al., 2011). At least two of them were preferentially expressed in the egg cell and, interestingly, their ectopic expression induced cell proliferation and activated an egg cell-*like* transcriptome. Members of the RWP-RK domain family contain the *MINUS DOMINANCE* (*MID*) factor, which is in the distantly related green algae species *Chlamydomonas reinhardtii* required for gamete differentiation (Ferris and Goodenough, 1997). Since the *RKD*-TFs of *Arabidopsis* are highly redundant and the genes are conserved over the plant kingdom, the single copy homologous *RKD*-TF in *Marchantia polymorpha* was used for functional analysis (Koi et al., 2016; Rövekamp et al., 2016). *MpRKD* showed wide expression in *M. polymorpha*, but preferentially high in antheridia, developing egg cells, and sperm precursor cells. Lines with downregulated expression showed large cells at the base of the archegonium, indicating that egg cell specification occurs on the bases of anatomy and position, however, in the absence of specific molecular markers (Rövekamp et al., 2016). These cells underwent cell divisions instead of entering the quiescent egg cell stage, suggesting a role of *MpRKD* in establishing and/or maintaining the quiescent state of the egg cell prior to fertilization. *MpRKD* mutants lacked effects on the overall morphology of reproductive organs, but showed striking defects in egg and sperm cell differentiation (Koi et al., 2016). Together, these results indicate that *RKD*-TFs are evolutionary conserved regulators of germ cell differentiation in land plants and particularly act in the gametophyte-to-sporophyte transition by preventing the egg cell from entering mitosis in the absence of fertilization, i.e., by suppressing parthenogenesis.

### The *FIS*-PRC2 and *RETINOBLASTOMA RELATED*1

Until the recent findings of *PsASGR-BBML* and *RKD*-TFs, a candidate for parthenogenesis in plants mentioned in literature was the *FIS-*PRC2 gene *MSI*1 (Köhler et al., 2003a; Guitton and Berger, 2005; section “Egg Cell Arrest and the Trigger for Embryogenesis”). It was isolated via a mutant screen in the sexual model *A. thaliana* and searched for because *MSI-like* homologs in yeast (*MSI*1) and mammals (*RbAp46/48*) were found to be involved in chromatin metabolism (Hennig et al., 2003). At that time, it became apparent that the modulation of chromatin structure played an important role in the regulatory decisions and gene expression during development, also in plants. As discussed above, the *FIS*-PRC2 is involved in gene suppression during seed development, particularly affecting the endosperm, whereas *MSI*1, and to a lesser extent also other *FIS*-class genes, affects embryo initiation in addition (Chaudhury et al., 1997). Other studies showed a role for the *FIS*-PRC2 in *balancing the maternal versus paternal gene dosage*, by showing plants with an increased maternal dosage resembling *FIS*-mutant phenotypes (Kradolfer et al., 2013). The results indicate that a release of gene suppression alone is insufficient to obtain viable seeds, but that this, particularly or solely, is a result of failure of the endosperm and maybe not embryo. Investigations on the role of the endosperm showed that, indeed, endosperm cellularization impacts embryo growth, and *FIS*-mutant embryos could be rescued on appropriate medium *in vitro* (Hehenberger et al., 2012). Also in other modes of *in vivo* induced parthenogenesis, such as via pollination with irradiated pollen or triploid inducer lines that results in haploid embryo development in some species (Germanà, 2006), embryos need to be rescued and cultivated *in vitro* due to failure of the endosperm. This indicates that release of the repressive state in the egg cell can be sufficient for the initiation of embryo development, however, finding clues for restoration of endosperm development is also necessary for successful parthenogenetic seed development.

The *FIS*-class genes *FIS*2 and *MEA* are imprinted genes. They are silenced throughout the life cycle of the plant, but become active in the female gametophyte, especially in the central cell, and remain expressed and active in the endosperm after fertilization, whereas the paternal alleles remain silent (Wang and Köhler, 2017). *MEA* is involved in the control of *embryo growth* in sexual species by repressing the maternal allele expression of the TF *PHE*1 (Köhler et al., 2003b). Thus, *PHE*1 is also imprinted, but expressed from the *paternal* allele only. A recent study asked the question what would happen with embryo growth in autonomous apomicts, where paternal alleles are absent (Kirioukhova et al., 2018). It was hypothesized that the silencing of maternal alleles might have become reduced or relieved during the evolution of apomixis, allowing maternally imprinted genes to be expressed from the maternal allele. This was tested for *PHE* in sexual *versus* asexual *Boechera*, a close relative of *A. thaliana*. In apomictic *Boechera*, the maternal *PHE-like* allele indeed was expressed, indicating a reversion of the imprinting status of this gene. In addition, a heavily methylated 3′MR was deleted from the *PHE*-alleles in apomicts, allowing increase of their expression. The authors proposed a model in which parthenogenesis in *Boechera* evolved via changes in epigenetic regulation of imprinted genes based on changes in DNA methylation (see Figure 3 in Kirioukhova et al., 2018). This shows parallelisms to an artificially induced case of parthenogenesis in mice through the loss of distal DNA methylation, resulting in maternal activation of the paternally expressed *Insulin-like growth factor* 2 (*Igf*2) gene (Kono et al., 2004). Thus, a modified role in transcriptional regulation of maternal alleles is indicated and interesting to further investigate in the context of parthenogenesis.

*FIS*-mutant phenotypes are resembled also by phenotypes of *RETINOBLASTOMA RELATED*1 (*RBR*1) mutants (Ebel et al., 2004; Johnston et al., 2010). This gene is related to the tumor suppressor gene *RB* in mammals, which has a role in inhibiting cell cycle progression. *RBR*1 in plants functions in cell cycle control during gametogenesis, with mutants showing supernumerary nuclei at the micropylar end and impaired cellularization (Johnston et al., 2008, 2010). Polar nuclei do not fuse in *rbr* gametophytes and cell-type-specific markers usually lack expression. *RBR*1 represses the G1/S-phase transition through inhibiting E2F transcription, and this, in turn, involves *RBR*1-phosphorylation that influences the *RBR*1-E2F interaction (Boniotti and Gutierrez, 2001; Kuwabara and Gruissem, 2014). Cyclin-dependent kinase (CDK) A combined with cyclin (CYC) regulatory subunit D (CDKA/CYCD; serine-threonine protein kinase) is involved in this phosphorylation. In interaction with *MSI*1, *RBR*1 also plays a role in the downregulation of *METHYLTRANSFERASE*1 (*MET*1) (Jullien et al., 2008). Reduction of *MET*1 in the central cell is essential for the activation of *FIS*2 and thus for the *FIS*-PRC2. Indeed, *FIS*2 expression is reduced in *rbr*-gametophytes (Johnston et al., 2010). Thus, *RBR*1 and genes associated to its functioning and/or to cell cycle progression are additional candidates to be involved in the suppression of spontaneous embryo and endosperm development and, as such, to have a role in parthenogenesis.

### Genes Involved in the Induction of Ectopic Embryogenesis

A number of other genes, mostly TFs, have been reported to be involved in the induction of embryogenesis ectopically and/or *in vitro* after overexpression (excellent reviews by Radoeva and Weijers, 2014; Horstman et al., 2017). They are mentioned here for completeness, but we refer to the other reviews for their listing and details, since a specific role in parthenogenesis is yet undetermined. Whether they are part of one or a few larger networks also needs further elucidation. Among them are *AP*2-TF family genes, including *BBM* (*AIL*2/*PLT*4) (see also section “The *PsASGR-BabyBoom-Like* gene”, above) most other *AIL*-genes, and *WOUND INDUCED DEDIFFERENTIATION* 1 (*WIND*1) (Horstman et al., 2017) that *support* embryogenesis. In addition, most genes of the “LAFL”-network, namely, *LEAFY COTYLEDON* 1 (*LEC*1), *LEC*1-Like (*L1L*), and *LEC*2, another member of the RWP-RK domain-containing family, *RKD*4 (see also section “RWP-RK Domain (RKD)-Containing Transcription Factor”), and the homeodomain TF *WUSCHEL* (*WUS*). There are also genes that function more indirectly by *increasing the capacity* for embryogenesis, such as *AGAMOUS-Like* 15 (*AGL*15) and *SOMATIC EMBYOGENESIS RECEPTOR KINASE* 1 (*SERK*1). Last, some genes are known to be involved in the *suppression* of embryogenesis, including the chromatin-helicase-DNA binding gene (*CHD*3/4)-*Like* chromatin remodeling factor encoded by *PICKLE* (*PKL*), genes of the PRC1 and -2 (see sections “Egg Cell Arrest and the Trigger for Embryogenesis” and “The *FIS*-PRC2 and *RETINOBLASTOMA RELATED*1”), and the *HIGH-LEVEL EXPRESSION OF SUGAR-INDUCIBLE GENES VAL*1 and *VAL*2. Yet knowing the putative role of parental expression of *Os-BBM*1 in embryogenesis, it is relevant to investigate this also for the other genes mentioned.

**FIGURE 2:**
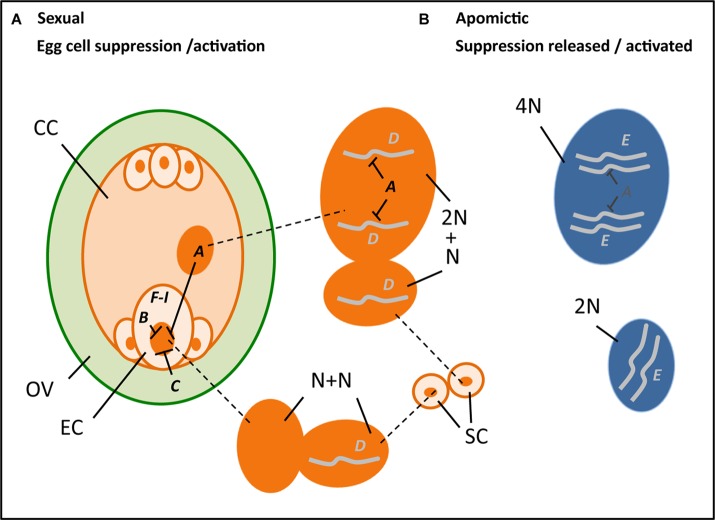
Hypothetical cartoon summarizing the potential processes and genes involved in parthenogenesis (***A–I***, with ---| denoting suppression) (see also Table 1), with *left* an ovule (OV, green) that encloses a reduced embryo sac (orange), including an egg cell (EC), central cell (CC), two synergids (next to the EC), and three antipodal cells, and outside it two sperm cells (SC, orange), in the middle the enlarged nuclei (dark orange) of the central cell (top, 2N; N = chromosome set after reduction division), sperm cells (middle, each N) and egg cell (bottom, N) at karyogamy, and *right* the unreduced nuclei (dark blue) of an apomictic central cell (top, 4N) and egg cell (bottom, 2N). ***A***, ***B***, and ***C*** denote genes involved in the *suppression of transcription in the egg cell*, particularly present in the central cell ***(A)*** notably the *FIS*-PRC2, egg cell ***(B)*** possibly *RKD*-TF, and the surrounding ovular tissue ***(C)*** potentially *AGO*9. ***D*** and ***E*** denote genes that *support embryogenesis* either after fertilization and transferring a paternally expressed, maternally silenced gene ***(D)*** as hypothesized for *Os-BBM*1, or via natural expression in an apomict ***(E)*** as is found for *PsASGR-BBML* and possibly involves reduced or absence of suppression. ***F*–*I*** denote genes and other stimuli that are found to be involved in embryogenesis either after *ectopic overexpression* to induce somatic embryogenesis ***(F)***, for example, *LEC*2, *increase embryogenesis capacity* ***(G)***, e.g., *SERK*1, being involved in *auxin response* ***(H)*** as recently found for *bHLH*49, or changes in *physiological conditions and abiotic stress factors* ***(I)*** such as Ca^2+^ and heat stress.

In summary, a *PsASGR-BBML* gene has been isolated and verified as the first parthenogenesis gene by demonstrating its functionality in related, sexual relative grasses such as pearl millet and rice, but not yet in eudicots. Other candidates and studies support that the suppression of spontaneous embryo and endosperm development in sexual reproduction is under tight epigenetic control and release of this control allows for the initiation of spontaneous embryo and endosperm development. This is shown to involve *FIS*-PRC2 genes and genes associated to it and to cell cycle control. Although initiated, mutants of these genes show early embryo arrest and endosperm development up to cellularization, indicating that a release of transcriptional suppression alone is not enough to obtain viable seeds. Functional endosperm is important in addition, either because of probable roles in the regulation of embryogenesis, but especially also to nourish the embryo. Restoring endosperm development is, therefore, necessary for successful seed development via parthenogenesis. Alternatively, the haploid embryos can be cultivated *in vitro* after embryo rescue, as is also done in some of the other haploid induction methods currently used in *DH*-production. Interesting recent results show that *Os-BBM*1 is paternally expressed, maternally silenced, and hypothesized to induce embryogenesis in rice egg cells after fertilization. Other recent results support a role for evolved changes in apomicts in this context, by showing the normally paternally expressed *PHE-Like* genes to be maternally expressed in apomictic *Boechera*. The results converge upon the importance of genes involved in the suppression of transcription and modifications thereof in apomicts at one hand and genes involved in the developmental process for which either transcription is allowed or artificially overexpressed on the other in parthenogenetic reproduction. In Table 1 and Figure 2 this convergence is summarized.

**Table 1:** Genes important in either the suppression or activation of embryogenesis *in vivo* or *in vitro* in plants and potentially relevant for parthenogenesis.

Activity	Type	Species	Sexual/apomictic	Gene family	Gene(s)	Expression pattern	Expressed in Egg Cell	Function	See Section
Suppression of Egg Cell	***A***	*Ath*	Sex	*PRC*	*MSI*1	Reproductive structures	(-)	Gene expression, DNA-methylation	“Egg Cell Arrest and the Trigger for Embryogenesis” and “The *FIS-PRC*2 and *RETINOBLASTOMA RELATED*1”
		*Ath*	Sex	*FIS-PRC*2	*MEA/FIS*1, *FIS*2, *FIE/FIS*3	*Maternal*, Central cell, Endosperm	-	Imprinted gene expression, DNA-methylation	“Egg Cell Arrest and the Trigger for Embryogenesis” and “The *FIS-PRC*2 and *RETINOBLASTOMA RELATED*1”
		*Ath*	Sex	*RBR*	*RBR*1	Reproductive structures	(+)	Nuclear prolifieration, imprinted gene expression	“The *FIS-PRC*2 and *RETINOBLASTOMA RELATED*1”
		*Ath*	Sex	*SWI/SWF, CHD*3	*PKL*	Ubiquitous	+	Embryogenesis, cell proliferation, chromatin	“Genes Involved in the Induction of Ectopic Embryogenesis”
	***B***	*Mpo*	Sex	*RWP-RK*	*Mp-RKD*	Ubiquitous, gamete (precursor)s	+	Germ cell differentiation, entering mitosis	“RWP-RK Domain (*RKD*)-Containing Transcription Factor”
	***C***	*Ath*	Sex	*RISC*	*AGO*9	Plant ovule	(-)	Non-cell autonomous TE-silencing	“Egg Cell Arrest and the Trigger for Embryogenesis”
Zygotic embryogenesis	***D***	*Osa*	Sex	*AP*2*/ERF, AP*2*L*	*Os-BBM*1	*Paternal*, sperm cell, zygote	-	Embryogenesis after fertilization or egg cell transgene expression	“The *PsASGR-BabyBoom-Like* gene”
Apomictic embryogenesis	***E***	*Psq*	Apo	*AP*2*/ERF, AP*2*L*	*PsASGR-BBML*	Unfertilized ovaries, embryos, anthers	(+)	Parthenogenesis	“Genetic Control of Parthenogenesis in Apomicts and Its Independency From Apomeiosis and Polyploidy” and “The *PsASGR-BabyBoom-Like* gene”
Somatic embryogenesis	***F***	*Ath*	Sex	*AP*2*/ERF, AP*2*L*	*AIL*2*/BBM/PLT*4 *, other AIL/PLTs*^1^	Late embryo, seeds	-	Cell proliferation, embryogenesis	“Genes Involved in the Induction of Ectopic Embryogenesis”
		*Ath*	Sex	*“LAFL”*	*LEC*1*, LEC*2*, L*1*L^1^*	(Early) embryo, seedling	-	Embryo development, maturation	“Genes Involved in the Induction of Ectopic Embryogenesis”
		*Ath*	Sex	*Homeodomain*	*WUS/PGA*6^1^	Shoot stem cell niche	-	Stem cell maintenance	“Genes Involved in the Induction of Ectopic Embryogenesis”
		*Ath*	Sex	*RWP-RK*	*RKD*4*/GRD^1^*	Early embryos	-	Embryo development	“Genes Involved in the Induction of Ectopic Embryogenesis”
Embryogenetic capacity	***G***	*Ath*	Sex	*RWP-RK*	*RKD*1*, RKD*2^2^	Egg cell and egg cell apparatus	+	Egg cell specification and differentiation	“RWP-RK Domain (*RKD*)-Containing Transcription Factor” and “Genes Involved in the Induction of Ectopic Embryogenesis”
		*Ath*	Sex	*MADS-box*	*AGL*15^2^	Embryo	+	Embryo development	“Genes Involved in the Induction of Ectopic Embryogenesis”
		*Ath*	Sex	*LRR-RLK*	*SERK*1^2^	Ovule, early embryo, vascular tissue	-	Embryo development, co-receptor	“Genes Involved in the Induction of Ectopic Embryogenesis”
	***H***	*Ath*	Sex	*bHLH*	*bHLH49*^3^	Early embryo	(-)	Mediating auxin response, dedifferentiating suspensor cells	“Egg Cell Arrest and the Trigger for Embryogenesis”


*^*1*^After ectopic and overexpression; ^*2*^in culture, *in vitro*; ^*3*^and bHLH-network.*

## The Use of Parthenogenesis in Plant Breeding

The ultimate goal of identifying a gene for *parthenogenesis* is to apply it in protocols for breeding line production in order to induce either *gametophytic* or *sporophytic embryogenesis* in a variety of cell types and plant species. This is of particular interest in the context of *DHs* production, a method that is widely used for the instant production of homozygous lines via haploid induction technology followed by chromosome doubling methods (reviewed by, e.g., Germanà, 2006, 2011; Belogradova et al., 2009; Islam and Tuteja, 2012; Hand et al., 2016; Ikeuchi et al., 2016; Horstman et al., 2017). Current methods include, among others, *in vivo* induction of spontaneous haploid embryo formation at (very) low frequencies via wide crosses, crosses with triploids, or by using irradiated pollen, usually followed by embryo rescue and *in vitro* embryo cultivation, and *in vitro* induction of embryogenesis in response to different (a)biotic stress factors. These methods, however, need time-intensive and species-specific protocol development and lack application in a number of important crops. Although successful in particular species or genotypes, others can be completely recalcitrant to produce *DH*s. Understanding the genetic basis of egg cell activation and the initiation of embryogenesis will largely contribute to the production of a wide variety of *DH*s.

Most interesting is the combination of *parthenogenesis* with various forms of modified meiosis. Particularly the combination with *apomeiosis*, the *omission of first meiosis*, resulting in unreduced gametes that maintain all or most of the genetic and epigenetic variation of the mother plant, is interesting in order to produce *clonal seeds*. This enables the maintenance of vigorous *F1-hybrids* that are usually produced via extensive crossing and careful selection procedures involving five or more growing seasons, however, segregate in the subsequent generation. Being able to re-grow valuable *F1-hybrids* over more than one generation has high potential for food security and the increasing demand on food. Proof-of-principle for synthetic clonal reproduction was obtained by Marimuthu et al. (2011) using either the *DYAD* mutant [Ravi et al., 2008; one allele of *SWITCH*1 (*SWI*1): Mercier et al., 2001; Agashe et al., 2002] or the “*turning MEIOSIS into MITOSIS*” (*MiMe)* variant (d’Erfurth et al., 2009) to obtain unreduced gametes in combination with the *CENTROMERE-SPECIFIC HISTON* 3 (*CENH*3) mutant (Ravi et al., 2011) to fertilize the central cell without genomic contributing to the embryo. This method is now awaiting improvements to produce unreduced gametes at high frequency as well as identify or produce *CENH*3-*Like* variants in crops. Another interesting modified meiosis variant is the *omission of second meiosis* to produce (near-)homozygous gametes, using mutants of *OMISSION OF SECOND DIVISION* 1 (*OSD*1) (d’Erfurth et al., 2009). This is particularly of interest in the (near-)*Reverse Breeding* approach in which (near-)homozygous parental lines and chromosome substitution lines are produced in one generation (Dirks et al., 2009). Reverse Breeding relies on the suppression of recombination during the first meiosis and omission of chromatid separation in the second. All these protocols need the induction of embryo development from the gametes produced and are awaiting capacity for *in vivo* or *in vitro* embryo induction. A third very useful application of parthenogenesis in plant breeding is the potential to link *diploid* with *polyploid gene* pools, in alternation with apomeiosis. This makes it, for instance, much easier to cross-in interesting characters of diploid wild relatives to the usual tetraploid crop varieties in potato. All three aims contribute to the control of plant reproduction and breeding and are highly relevant in order to optimize crop development and increase plant productivity.

## Conclusion and Future Perspective

This review provides a summary of current knowledge on parthenogenesis in plants obtained from studies in natural apomicts and mutants in sexual model species. Several lines of evidence from natural apomicts support that parthenogenesis inherits and functions independently from apomeiosis and endosperm formation, and that it is a monogenic and dominant trait. Results also show that parthenogenesis is expressed and active in the egg cell and independent from signals from the surrounding sporophytic tissue. Parthenogenesis functions normally in reduced di-haploid egg cells, resulting in viable embryos that grow into di-haploid plants. It also works in *true* haploid eggs, but with (much) lower frequency in producing viable plants and with the haploid offspring usually being infertile, and in aneuploid eggs, although with early embryo abortion. The reason that parthenogensis is usually absent from haploids in nature is indicated to be the result of a linked deleterious genetic load. The relationship of parthenogenesis to the central cell and endosperm is more complicated. The central cell plays a role in egg cell suppression in sexual reproduction (see Figure 2), and functional endosperm is needed for successful embryo development. It is not entirely clear if the parthenogenetic egg cell also undergoes a (short period) of quiescence in which the chromatin is repressed and transcription is silenced. These processes might be necessary for obtaining *totipotency* in the zygote, but has to be confirmed. Studies in sexual model systems, particularly Arabidopsis, support that the *release* of egg cell repression and transcriptional silencing results in the initiation of embryo development. Genes that are involved in this, particularly genes of the *FIS*-PRC2 or related to cell cycle control, e.g., *RBR*1, and the recently uncovered *RKD*-TFs, may therefore have changed or become ineffective in apomicts. A possible mechanism for this could be a change in the target gene sequence by which the silencing is reduced, e.g., by a deletion of a region involved in heavy methylation as was found in *PHE*-alleles in apomictic *Boechera* (Kirioukhova et al., 2018). Similarly, the functioning of *PsASGR-BBML* in apomicts, and the maternal silencing of the related *Os-BBM*1 in sexual rice, may hint to a reduction of maternal silencing of *BBM-Like* in apomictic grasses. The release of gene suppression particularly in the *FIS*-PRC2 mutants also affects the central cell, leading to spontaneous endosperm development up to cellularization. Embryos arrest at an early stage in these mutants, and this may also involve failure of the endosperm, since they can be rescued by *in vitro* cultivation. Embryos obtained with *PsASGR-BBML* also need either embryo rescue or fertilization of the central cell in order to allow endosperm development and embryo growth progression. The sexual endosperm is maternally to paternally genome dosage-dependent in most species (*2m : 1p*), whereas in apomicts, this dosage is usually highly disturbed without any obvious effect. Apparently, apomicts have evolved several mechanisms to overcome these requirements. One study supports that the function of the *FIS*-PRC2, and thus transcriptional silencing, can be suppressed by an increase of the maternal dosage in the endosperm, leading to a *FIS*-mutant-like phenotype. Whether this mimics the situation in the central cell of apomicts has to be resolved (see Figure 2). Altogether, the results show that parthenogenesis involves changes in epigenetic regulation needed to allow genes that are essential in embryo induction to be expressed from the maternal allele. They also show that parthenogenesis functions independently from endosperm development, but that the absence or failure of the endosperm impacts successful embryo and plant development. Insight into the mechanisms that have developed in apomicts to overcome the failure of the endosperm, and the development of methods to restore endosperm production, for example, through artificial crosses to fertilize the central cell, is needed and is the next challenge in successful seed production via parthenogenesis.

In the near future, establishing the function of the emerging parthenogenesis candidates in a wider sense is one of the main priorities as is the identification and/or engineering of parthenogenesis in non-grass species. A second priority is to transfer the knowledge to crop species to make it useful in plant breeding and in *in vitro* embryogenesis protocols. Third is to improve the experimental separation between the functioning of the egg cell and embryo growth progression on one hand and the dependence of the central cell and endosperm on the other in research on parthenogenesis, and to further unravel the mechanisms that underlie spontaneous endosperm formation. Fourth, to combine parthenogenesis with the different forms of “*omission of meiosis*” in order to use it as a tool in plant breeding, e.g., for clonal seed production/apomixis. Also interesting is to further unravel other mechanisms that have evolved in apomicts, such as the possible differences in egg cell quiescence as well as the ways in which the endosperm overcomes the maternal to paternal dosage requirements.

## Author Contributions

KV designed and wrote the manuscript. PO-A wrote some parts of the manuscript. PO-A and MS gave valuable review comments.

## Conflict of Interest Statement

The authors declare that the research was conducted in the absence of any commercial or financial relationships that could be construed as a potential conflict of interest.

## Abbreviations

ASGR: apospory-specific genomic region
DHs: doubled haploids
FIS: fertilization-independent seed
MZT: maternal-to-zygote transition
PRC2: polycomb repressive complex 2
TEs: transposable elements
TF: transcription factor
ZGA: zygotic genome activation
